# European Cohorts of patients and schools to Advance Response to Epidemics (EuCARE): a cluster randomised interventional and observational study protocol to investigate the relationship between schools and SARS-CoV-2 infection

**DOI:** 10.1186/s12879-022-07947-6

**Published:** 2023-01-03

**Authors:** Sara Raimondi, Sara Gandini, Gibran Horemheb Rubio Quintanares, Ana Abecasis, Pier Luigi Lopalco, Oriana D’Ecclesiis, Susanna Chiocca, Elisa Tomezzoli, Ilaria Cutica, Davide Mazzoni, Nuno Amparo, Marta Pingarilho, Daniela Carmagnola, Claudia Dellavia, Gianvincenzo Zuccotti, Chiara Ronchini, Federica Bellerba, Felix Dewald, Rolf Kaiser, Francesca Incardona, The Eucare WP4

**Affiliations:** 1 Department of Experimental Oncology, IEO, European Institute of Oncology IRCCS, Milan, Italy; 2 Institute of Virology, University Clinics of Cologne, Cologne, Germany; 3 Paul Ehrlich Institut, Langen, Germany; 4 Infectious Diseases Department, Instituto Nacional de Ciencias Médicas y Nutrición Salvador Zubirán, Mexico City, Mexico; 5 Global Health and Tropical Medicine, Institute of Hygiene and Tropical Medicine, New University of Lisbon, Lisbon, Portugal; 6 Department of Biological and Environmental Sciences and Technology, University of Salento, Lecce, Italy; 7 Applied Research Division for Cognitive and Psychological Science, European Institute of Oncology (IEO), IRCCS, Milan, Italy; 8 Department of Oncology and Hemato-Oncology, University of Milan, Milan, Italy; 9 Public Health Clusters’ Public Health Unit of Central Alentejo, Lisbon, Portugal; 10 Department of Biomedical, Surgical and Dental Sciences, University of Milan, Milan, Italy; 11 Pediatric Department, “Vittore Buzzi” Children’s Hospital, Milan, Italy; 12 Department of Biomedical and Clinical Science, University of Milan, Milan, Italy; 13 IPRO-InformaPRO S.R.L., Rome, Italy; 14 EuResist Network, Rome, Italy

**Keywords:** Schools, Pandemic, COVID-19, Children’s health, Adolescents’ health

## Abstract

**Background:**

Contradictory results were reported on the role of school closure/reopening on the overall SARS-CoV-2 transmission rate, as well as on which kind and level of mitigation measures implemented in schools may be effective in limiting its diffusion. Some recent studies were reassuring, showing that opening did not increase the community spread, although teachers and families are worried about the high class density. On the other hand, distance learning was associated with a negative impact on learning, sociability and psychological health, especially in vulnerable children. As it becomes clear that the SARS-CoV-2 pandemic will last for a long time, there is a high need for studies and solutions to support safe schools opening based on scientific evidence of harms and benefits. The Lolli-Methode (LM) is a strategy for epidemiological surveillance and early intervention aiming at SARS-CoV-2 outbreaks’ reduction in schools, relying on polymerase chain reaction analysis of saliva samples.

**Methods:**

In this cluster randomised trial protocol, we aim to determine whether the LM is useful to support schools opening and to reduce clusters and attack rates in schools, compared with the standard of care (SoC) surveillance by public health departments. This multicenter study will enrol 440 classes (around 8800 students, teachers and other personnel) from two countries, cluster randomised to LM or SoC. The samples from the pools will be collected and tested using PCR-based techniques. Test results will be combined with questionnaires filled in by children, parents, schoolteachers, and principals, concerning ongoing mitigation measures, their perceived psychological impact and other health and socio-economic information. An ancillary observational study will be carried out to study the prevalence of SARS-CoV-2 in schools, frequencies and size of clusters and attack rates, to compare the effectiveness of the different preventive measures adopted and to evaluate psychological issues in students and teachers in relation to the pandemic’s containment measures.

**Discussion:**

By the end of this study, we will have defined and characterised the applicability of the LM for SARS-CoV-2 surveillance, as well as the impact of pandemic preventive measures on children and teachers.

*Trial registration* International Standard Randomised Controlled Trial Number: NCT05396040, 27.05.2022*.*

**Supplementary Information:**

The online version contains supplementary material available at 10.1186/s12879-022-07947-6.

## Background and rationale

School closures represent a widespread non-pharmacological intervention (NPI) in the context of the current COVID-19 pandemic. The rationale for such a NPI has mostly been drawn from the reported beneficial effect of school closure during influenza pandemics. However, the COVID-19 disease spread and virulence by age have soon proved to be very different from those of influenza, with less frequent infections and milder manifestations in children than in adults [1–4]. Also, several studies showed that, when appropriate mitigation measures are implemented, the prevalence of positive cases in schools is lower than in the general population and the number and size of clusters are smaller [5–11].

This is indeed what emerged also from our cross-sectional and prospective cohort study in Italy, one of the European countries with the highest SARS-CoV-2 incidence, during the second COVID-19 wave (from September 30, 2020, until February 28, 2021) [12] on 7976 public school institutes (97% of total), accounting for 7,376,698 students, 775,451 teachers and 206,120 non-teaching staff members. The analysis also evidenced that school opening was not related to the second COVID-19 wave in Italy.

On the other hand, the recent epidemic variants Delta and Omicron, in combination with the low vaccine coverage of children, resulted in a significant increase of cases in children from 0 to 17 years of age, especially in the groups 0–6 and 14–17 years old. Some modelling studies showed a relevant effect of school closure/reopening on the overall transmission rate depending also on the impact on the overall mobility, although the authors underlined that their studies were not able to disentangle the results by the kind and level of mitigation measures implemented in the schools [13, 14]. Furthermore, there is little evidence and even less agreement on which mitigation measures are more efficient and as to what level of mitigation is needed [15, 16].

Most schools of all grades have been kept closed in many countries, preventing in-person learning and consequently generating an acute debate in society and science, which is still one of the most contentious issues of the COVID-19 pandemic. The spread of lineage Variant Of Concern (VOC) B.1.1.7 has worsened the fears and caused further schools’ closure in the UK and in Europe [17–20].

Importantly, school closure, supported or not by remote teaching, has been proven to cause harms to children and society in terms of health [21], psychological consequences [22, 23] and learning deprivation [24] with consequent long-term learning losses [25, 26], that are higher in socio-economically disadvantaged individuals [27] and that many consider to outweigh the benefits [19].

There is a high need for studies and solutions to support safe schools opening during a pandemic based on scientific evidence of harms and benefits. This means for instance to establish affordable and reliable surveillance systems to evaluate the prevalence, attack rates and clusters of SARS-CoV-2 in schools in relation to VOCs, to assess the best preventive measures to be adopted in schools in relation to VOCs, to disentangle the effects of school attendance (including related mobility) on the transmission rate of SARS-CoV-2 in the society as well as to evaluate the psychological health and learning damages associated to schools closure.

### The Lolli-Methode

Molecular salivary testing for the early detection of SARS-CoV-2 infection has proven to be a reliable tool for active surveillance and its use in communities has been reported [28]. The Institute of Virology at UniKoeln has implemented its use and developed a non-invasive SARS-CoV-2 screening method and intervention strategy based on saliva pool testing, namely “The Lolli-Methode” (LM) (exported with the name “Lolli Strategy” in Mexico) [29].

LM relies on saliva self-sampling by participants (adults and children, including babies) who need to suckle a swab as if it was a lollipop, for 30 s. Its application in schools implies that the swabs are pooled in defined groups, such as all the pupils and teachers of a class. Each pool/class is analysed collectively by a single PCR test. Negative pools imply no further action is needed. In case of a positive pool, all the individuals included in the group will have to collect again their saliva that, at this time, will be analysed individually.

The sampling/testing is meant to be performed twice a week, to allow early infection detection, competing with the time required by contact tracing protocols for the identification of positive cases and epidemiological surveillance purposes. Assuming a low positive rate in schools, this methodology is significantly more affordable in terms of logistics and costs with respect to separate analysis of individual swabs.

The sensitivity of LM was evaluated and found comparable with throat swabs plus nasopharynx swabs, both comparing single LM sample against swab and comparing single LM samples against pooled ones [29].

LM was introduced in pilot programs for children in kindergartens (“Kiko”) and schools, “B-FAST” [30], and “SCHOCO”, and extended, in collaboration with the Department of Health and the Department for Children, Youth and Family of the City of Cologne, in 32 kindergartens and 20 schools of Cologne.

LM was subsequently further extended to all the kindergartens (690) in Cologne, 130 kindergartens in Düren, and in North-Rhine-Westphalia state were included 95 kindergartens and all the schools (3700). In Mexico, the program has been successfully implemented as a pilot project in 394 schools and kindergartens of the Mexican state of Tabasco. Such accomplishments prove that LM can be adopted in different territories, populations, and conditions.

The LM design is intended to facilitate the implementation in any laboratory; therefore, any standard commercially available laboratory material can be used. The needed materials are: (i) swabs for sample collection, (ii) 50 ml capacity tubes for collecting the swabs in pools, and (iii) PBS or saline solution to hydrate the collected pool. In case that the recommended material (swabs, tubes) are not available, the material can be replaced by other commercial swabs or tubes which are equivalent. Standard material, namely Lolli-QM is available to verify the usability.

Specific indications on the materials and procedures are reported in the Additional file 1.

In the pilot phase in Cologne, 11 positive cases were detected in 32 kindergartens over a period of six weeks. The implementation in North-Rhine-Westphalia was conducted for 23 weeks with 2 vacation periods of 6 and 2 weeks, respectively, testing 742,771 individuals twice per week. The positivity rates were higher directly after vacation and were decreasing progressively after Lolli implementation [29]. The positivity rate at week 19 was 0.46%, reducing to 0.05% on week 26. After summer holidays we found 2.61% positivity rate at week 33 reducing to 0.92% three weeks later [29]. LM was implemented on a voluntary basis and acceptance resulted in more than 90%. No accidents or harm to any participant were reported or detected.

Comparator is the SoC, usually consisting in contact tracing activity performed by public health departments and routine surveillance based on symptoms prompting testing during large waves of the epidemic. It’s worth mentioning that SoC may undergo changes along the study, given that public health surveillance guidelines have been changing frequently along the pandemic.

The benefit/risk ratio for participants is very high, as there are potentially no risks coming from the performed tests. In fact, LM testing is well established and safe to perform. Moreover, it is less invasive than nasopharyngeal swabs and easily accepted by children. The benefits of this study consist in the possibility to find a more effective way to control the spread of the infection in schools compared to SoC and at the end reduce the number of days in distant learning. Furthermore, it contributes to provide a better understanding of the COVID-19 epidemics in the school setting.

## Study design

### Study objectives

#### Overall objective

The project represents a multidisciplinary interconnected effort to provide robust, data driven evidence to deal with SARS-CoV-2 variants and COVID-19 epidemics in the school setting, by comparing testing and containment strategies in different countries. Here we present the protocol version 1.1 from the 24.02.2022.

#### Primary objective

The primary objective is:

- To determine if regular screening with pooled saliva tests by LM is useful to support school opening and to reduce clusters and attack rates in schools, compared with SoC contact tracing by public health departments and surveillance based on symptoms prompting testing.

#### Secondary objectives

The secondary objectives are:

- To determine the incidence and prevalence of SARS-CoV-2, as well as clusters’ frequencies and size and attack rates in schools with the new variants, compared with the same indexes observed in the previous waves.
- To verify whether positive cases at school were caused by transmission within the school.
- To compare the effectiveness of different preventive measures adopted in different schools taking into account variants and vaccination (including social distance, wearing masks, preventive measures during lunch time, use of bubbles, extra-scholar activities).
- To compare the efficacy of different protocols of contact tracing in controlling clusters and attack rates and rates of quarantines.
- To investigate the potential psychological impact of the use of molecular saliva tests, different preventive measures and distant learning during schools' closures or quarantines in students, teachers and other school personnel, taking into account their socio-economic status and the household composition (single parent, presence of elderly in the house, etc.).
- To investigate differences in SARS-CoV-2 incidence by socio-economic status and distance from school/mobility issues.
- To investigate household secondary transmission attack rates.
- To evaluate schools usefulness as sentinel testing sites for community detection and control of SARS-CoV-2 epidemics.

### Study outcomes

#### Cluster randomised interventional study

##### Primary outcomes

- Frequencies of clusters in classrooms in which pooled saliva tests (LM) are performed in association with standard contact tracing protocols, compared with classrooms in which SoC only protocols are applied.
- Number of classes and of students in the classes that accept to participate in the LM study, if asked, during time.

##### Secondary outcomes

- Transmission/prevalence of SARS-CoV-2 in schools by variants, by assessing the proportion of students/staff with positive SARS-CoV-2 test identified by pooled saliva tests (LM).
- Size of clusters and attack rates in classrooms in which pooled saliva tests (LM) are performed, compared with classrooms in which standard contact tracing protocols is applied.
- Number/size of clusters associated with the use of different preventive measures in different classrooms, by prevalence of different variants and taking into account vaccination rates.
- Number of students participating in the project per class across time.
- Proportion of classes who accept to participate in the LM.
- Psychological status of students (in terms of emotional and behavioural problems) and school staff (in terms of of psychologcal well-being) through standardized questionnaires and ad hoc questions, from kindergarten to high school during the COVID-19 pandemic with respect to the use of LM and other preventive measures such as face masks, social distancing, distance learning, vaccines, etc., as well as attitude toward vaccinated and non-vaccinated students.Household secondary attack rate and dynamics of infections in terms of school imported or house imported, extending the LM to the household members of positive school cases.
- Social and mobility outcomes in terms of mobility changes and social interaction/distancing changes during the different waves will be analysed through statistical analyses and spatio-temporal analyses.

#### Observational study

##### Primary outcome

- To evaluate frequencies and size of clusters and attack rates in schools, compared with incidence, prevalence, frequency of SARS-CoV-2 positive subjects and clusters in the different waves.

##### Secondary outcomes

- To evaluate the psychological health of students and teachers during the COVID-19 pandemics: negative and positive feeling during confinement, behaviour and emotions experienced with respect to preventive measures (masks, social distancing), distance learning, and vaccines. The evaluation will be carried out through standardized questionnaires and ad hoc questions.Number/size of clusters by the usage of different preventive measures adopted in different classrooms by prevalence of different variants and taking into account vaccination rates.
- Social distancing practices changes along the pandemic and potential association with incidence.
- Mobility practices changes along the pandemic and potential association with incidence.

### Study design

#### General study design (Fig. 1)

**Fig. 1:**
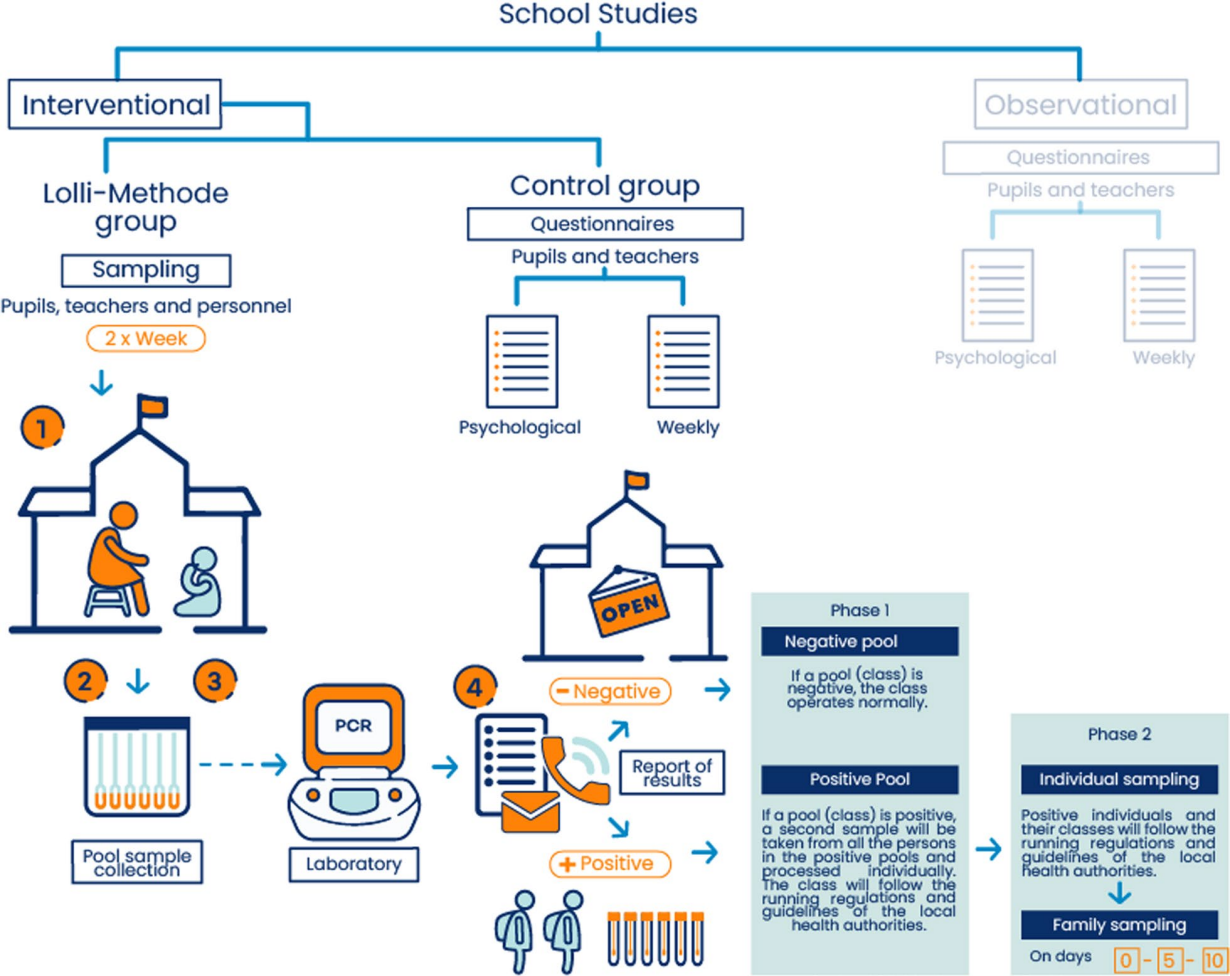
Study flowchart. The flowchart summarize the general study where is divided in two sub studies, (i) interventional study which contains a Lolli arm and a control arm; and (ii) observational study. A detailed description have been written in the main text. We want to acknowledge Pez Beta Design for the elaboration of the figure

A multicenter cluster randomised interventional study will be set-up to evaluate the efficacy and applicability of the LM. The study will enrol 440 classes (around 8800 students, teachers and other personnel). Statistical units are represented by classes, randomized to LM or SoC.

The study will be carried out for 6 months, from October 2022 to March 2023 and includes two saliva tests a week. We assume that in the course of the investigated period the incidence rate of SARS-CoV-2 infection will change and this will allow us to test our hypotheses under different epidemiological conditions.

The randomisation will be applied as cluster randomisation: classes will be randomised in each school to the adoption of the LM or to SoC with contact tracing activity by public health departments and regular surveillance based on symptoms indicating testing. The two groups of classes will be compared in order to assess whether the LM will reduce the frequencies of attack rates/clusters dimension compared with classes on SoC. Logistics and costs affordability will also be evaluated.

An observational retrospective and prospective study will be carried out to investigate the prevalence of SARS-CoV-2 in schools with different variants, frequencies and size of clusters and attack rates in schools, the effect of different NPIs on SARS-CoV-2 diffusion in the schools and the related psychological effects. The study will involve classes/schools that are not involved in the interventional randomised study.

### Study population

Kindergartens and school-age children, school personnel and children’s household members.

Schools of all grades have been considered, involving students and all school personnel in order to evaluate the LM in a real environment for its possible deployment and to understand the dynamic of the spread of SARS-CoV-2 infection in the schools.

In case of positive pools, family members of positive individuals will be tested as well, in order to analyse the possible use of schools testing as a sentinel to support the contact tracing analysis, and to evaluate whether the students or school personnel have been infected at school or within their households.

#### Inclusion criteria

- Kindergartens and school-age children from public or private schools.
- Enrolled children may be students of classes that have expressed their consent to participate (no limit of participants per class is adopted).
- School personnel of participating schools.
- Household members of participating children/students.
- Informed consent (for minors the informed consent will be signed by parents or legal guardians).
- For minors: willingness to participate.

#### Exclusion criteria

- No informed consent by schools or children, or the adult participant.
- Suspicion of acute COVID-19 infection:
  - In case of unknown respiratory infection, no presence of symptoms that can be attributed to SARS-CoV-2 for at least 48 h.
  - In case of confirmed SARS-CoV-2 infection: exclusion for at least 21 days from PCR-positive diagnosis and no presence of symptoms for at least 48 h.

#### Recruitment and screening

Schools will be selected in collaboration with public health authorities. Letters will be sent to principals, explaining the objective of the two studies and evaluating the interest of schools to participate in one or both the studies.

Once schools have declared their interest to participate, an informative session will be organized with school personnel and representatives of the families and students. Material describing the characteristics of the study will be delivered to the families to explain aims and procedures. Families and students will be asked to sign an informed consent form. Information will be available at a dedicated web page within the EuCARE website (https://eucareresearch.eu).

For the cluster randomised interventional study, classes will be randomized to one of the two groups. At the beginning of each testing session, participants will be screened for exclusion criteria.

#### Assignment to study groups

Classes will be assigned to the study arms with a randomised web-based procedure: the web randomisation module will accept as input the school-class identifier and will provide as output the study arm, based on a randomisation list balanced by four.

The randomisation will be stratified according to age classes (e.g. kindergarten age; primary intermediate school 6–13 years; secondary school).

#### Criteria for withdrawal/discontinuation of participants

A participant will be excluded from the study when one of these conditions occurs:

- withdrawal of the consent at any time of the study.
- evidence of serious adverse events or accidents incompatible with study continuation.

Furthermore, a subject may be excluded by the study cohort, following autonomous decision of the responsible PI, for any reason or consideration made in the interest of the subject itself or of the classroom.

### Study intervention

#### Identity of investigational products

##### Experimental intervention

The LM is a screening strategy based on molecular analysis for SARS-CoV-2 of pooled saliva samples taken from a group of individuals, in our case a school class including the teacher present in the class at that time. If the pooled sample is positive, then each class students is tested individually and all negative tested students/teachers are permitted to return to school. If the positive is identified, this subject will stay home according to the directions of the health authorities.

The procedure adopts standard material (PCR, swabs, plastic tubes for collection of swabs).

The pooled sample is treated as follows: all swabs from one class are collected in a single Falcon tube. As the Falcon tube enters the lab, 3 ml of PBS will be added to it. After mixing, a portion of the sample will be used for testing. Testing will be performed using standard SARS-CoV-2 PCR diagnostic approaches available in each laboratory.

The samples are collected at the first school hour in the morning. The results of the molecular analysis are provided in the same day. In case of a positive pool, individual saliva re-testing of each class member is performed the day after to identify the positive individuals.

The remaining sample of each positive individual will be stored for long term conservation to further analyse variants by punctual mutation PCR test or by sequencing.

##### Control intervention

The control arm will undergo conventional surveillance based on symptoms prompting testing and contact tracing according to SoC, to identify SARS-CoV-2 positive students, under the supervision of dedicated personnel (local health authorities personnel and/or school personnel). The local PIs and staff will be available to provide support for any questions.

#### Administration of experimental and control interventions

##### Experimental intervention

The children in the experimental group will undergo regular screening for the whole study periods using the LM. This consists of PCR pooled saliva tests as previously described. The main specificity of the approach is that each class is considered as a unit of testing and therefore tested in a single PCR. The teacher present in the class at the time of the test and the school personnel “adopted” by the class will participate in the pool test together with the class. Each pool will include the students, the teacher at the time of sampling and the auxiliary school staff associated to each class.

We will propose the pooled saliva test to each class participating in the study twice a week.

Specifically, each involved person will suck on a swab for 30 s to collect saliva. All swabs from each class will be collected in a single Falcon tube. The tubes of all the participating classes are stored at a safe place in the school and then collected and transported to the lab.

If a class (pool) tests positive, results will be reported to the schools and parents later that day and individuals whose sample were within the positive pool will be re-tested individually the next morning.

The results of the individual tests are provided within the same day or exceptionally the day after. The positive results are transmitted to the relevant health authorities and have the related legal value (involved laboratories are certified, approved laboratories).

The positive subjects and the whole class/school will follow the containment measures foreseen by the national and regional regulations applicable in each country at each moment.

Householders of a positive child will be tested as soon as the child is known positive (day 0), at day 5 and at day 15. The affected householders collect their single swabs at the agreed time points and store them to be analysed after quarantine (retrospectively). This will allow us to determine the transmission route in each family as well as secondary attack rate.

The usual surveillance based on symptoms indicating testing and contact tracing activity will continue according to SoC in the interventional arm too.

Remnants of the samples can be used for re-analysis for other pathogens. This will only be performed after the study group decides to perform such additional analysis. This will be performed retrospectively and anonymously.

##### Control intervention

The classes in the control group, including the assigned teachers and school personnel, will follow SoC countries measures regarding surveillance based on symptoms and contact tracing. The usual surveillance will be conducted by the personnel devoted to it in SoC situation. Study personnel from the involved PIs will be available to support.

### Study assessments

#### Assessments of outcomes

##### Assessment of primary outcome

The frequency of clusters in classrooms will be evaluated. We will compare classrooms where pooled saliva tests (LM) are performed in association with standard contact-tracing protocols, with classrooms in which only standard contact-tracing protocols are applied. The transmission/prevalence of SARS-CoV-2 in schools will be also evaluated, by assessing the proportion of students/staff with positive SARS-CoV-2 test identified by pooled saliva tests (LM).

##### Assessment of secondary outcomes

Comparing SoC with LM, the size of clusters and attack rates in classrooms will be evaluated.

The collected samples will be analysed via each available method for variants detection (for instance: differential PCR /real time PCR/ whole genome sequencing).

The effectiveness of different preventive measures adopted in different schools will be compared. Different types of protocols regarding contact tracing will also be compared.

Secondary transmission attack rate in households will be assessed for positive cases detected by pooled LM. The same assessment will be applied to the study of the SARS-CoV-2 spread dynamics in schools.

Simultaneously with the evaluation of the efficacy of LM, a psychological evaluation of the status of children during the COVID-19 pandemic will be performed (negative and positive feelings during confinement, worries and expectations about school resumption). Data will also be collected in order to study the correlation between environmental or/and temperamental factors and the stress levels reported by the children. For this goal, self-reported quality of life questionnaires will be implemented.

Mobility information and non-pharmaceutical interventions that were enacted in the school's area will be collected and evaluated as potential effect modifiers. This will be done using data collected in public databases, as well as data curated by the local PIs.

##### Assessment of safety outcomes

All safety concerns regarding the usage of the LM will be explained to the kids and their tutors, and every question will be answered at the beginning of the study.

During the intervention, any safety concern of this procedure will be collected via the school director and workers. All situations reported will be taken into consideration regarding that participant’s continuation or premature stop from the study.

#### Procedures at each visit

The Lolli will be performed two days a week, the “Lolli days”. The procedure can be managed autonomously by the schools or with support by an external person from the research centre.

Each Lolli day, the students who participate in the trial are given a swab at the entrance in class. They suck the swab for 30 s and then they all enter their swabs into the same large tube for the class. The teacher and the non-teaching personnel do the same.

The teacher puts a label with the class ID and barcode on the tube and enters into the eCRF the information of questionnaire T1 (date, class identifier, n. of absent pupils, n. of pupils who refused to carry out the test).

The responsible person for each school collects the tubes and puts them into a plastic bag.

The contracted courier authorised to biological samples transportation will then arrive at agreed time and will bring the plastic bag containing all the tubes to the laboratory. Tubes will be sent to the study laboratory in the morning. Results will be returned to the monitor in the same day.

If the pool is positive, the monitor will contact the school and the Public Health authorities in the same day and request the positive class participants to return to school the day after to collect the Lolli test for the individual testing. While waiting for the individual results, the tested participants will return back home or go to school according to the national regulation in force.

The individual results will then be communicated to the relevant public health authorities who will conduct the epidemiological investigation supported by the study questionnaire and will assign the containment measures according to the regulation in force.

The procedures set in place to accomplish the objectives of this project will be monitored during the entirety of the study by the PIs of the centre that enrolled the school.

### Statistical methods

#### Hypothesis

The main hypothesis is that the LM can reduce the frequency of clusters (i.e. two or more cases in a class within a scholar week).

The main endpoint is the frequency of cluster per class in a scholar week.

Secondary endpoints are cluster size and duration of consecutive/serial infections in the same class.

#### Determination of sample size

Clusters are defined as epidemiologic links between an index patient and one or more persons who likely acquired SARS-CoV-2 infection in class (i.e., class-associated cases). Given the average infection rates in contacts of about 4% per class [31], a sample size of 440 classes (220 per arm) achieves 80% power to detect a difference between the group proportions of 3.5%. The proportion in arm with LM is assumed to be 4% under the null hypothesis and 0.05% under the alternative hypothesis. The test statistic used is the one-sided Z test with pooled variance with a 5% significance level.

#### Statistical criteria of termination of trial

There is no statistical criteria for termination of trial. An interim statistical analysis will be performed after the first three months, but given the frequent changes of the epidemiological situation, the final statistical analysis (after six months of intervention) will be performed independently by results of the interim analysis.

#### Planned analyses

##### Datasets to be analysed, analysis populations

The analysed population consisted of pupils and students of kindergartens, primary and secondary schools located in Italy, Portugal, Germany and Mexico.

##### Primary analysis (LM evaluation trial)

The number of clusters per class per week will be compared between the two arms: LM + SoC arm vs SoC only. Absolute and relative frequencies of clusters will be reported by arms and compared at univariate analysis with Chi Square or Fisher exact test, as appropriate. Univariate analysis will be performed and the p-values for statistical tests of associations of categorical and continuous variables with the two treatment arms will be reported (Chi square/Fisher exact test and T-test/Wilcoxon sum rank test for categorical and continuous variables, respectively). We will also investigate the psychological impact of Lolli tests and preventive measures to reduce the risk of infections; we will compare the frequency of infections according to socio-economic status, regions, teacher vs student, educational level, also taking into account vaccination rates within each class.

##### Secondary analyses (ancillary observational study)

Univariate and multivariable analyses will be carried out to identify other independent factors associated with the presence of clusters per class per week (yes vs no) with logistic regression models, adjusting for possible confounding variables. Individual infection (infected vs/not infected per week and in the whole study period) will be also evaluated with logistic regression models, adjusting for possible confounding variables and also comparing teachers and students. Finally, we will evaluate time from the trial start to first cluster with survival analyses.

Random effects models for repeated measures will be carried out to investigate independent factors associated with psychological scores, taking into account of confounding factors such as age, sex, vaccination status, socio-economic status, different use of preventive measures, and extra-scholar activities.

##### Artificial intelligence (AI) based analysis

An evaluation will be carried out to study if and how NPIs applied within the vicinity of the schools in the EuCARE study, variations in mobility and variables that might be linked to the disease spread have affected the outcomes of the study. This will be answered by evaluating treatment bias within the trial and between the control SoC arm and the prospective study.

We will stratify the results by the potential effect modifiers—different class characteristics, NPIs, variation in mobility and variables related to disease spread and evaluate their influence on the study results.

Additional analysis will check whether the outcome of the school studies can be predicted from the mutation prevalence information, the preventive measures and population data during the trial and if yes, what can be learned from these associations. This will be accomplished by training a prediction algorithm taking the class characteristics, mutation prevalence, preventive measures and whether the LM was applied or not, and predicting the number and size of infection clusters during follow-up. If such a model would result with satisfying accuracy levels, then a post-hoc analysis for interpreting interaction associations between the measures included in the model will be provided and an importance analysis on the model features will be executed.

##### Handling of missing data and drop-outs

Since the number of clusters may depend on the number of tests per each class, we will collect information on the number of students who agreed to be enrolled in each class, each week, and we will take into consideration this information in the statistical analysis, adjusting and stratifying for the response rates.

## Discussion

The EuCARE consortium involves 18 partners from 10 different countries and has been funded by the European Commission in the frame of the research aimed at fighting SARS-CoV2 virus and its variants, in the context of the EuCARE project, “European Cohorts of Patients and Schools to Advance Response to Epidemics”. The project’s ultimate goal is to find solutions to safely support school reopening within the COVID-19 pandemic on the basis of scientific evidence, taking in consideration the impact that a prolonged school closure can have on education, wellbeing and personal development of children.

As detailed in the website (https://eucareresearch.eu/studi-sulle-scuole), the study -which involves a multidisciplinary group of scientists- is made of two parallel sub-studies: an observational and an interventional study, to evaluate epidemiological and psychological aspects, prevention measures and efficacy of the saliva-based “Lolli-Methode” for COVID-19 screening. The “Lolli-Methode” is based on the collection of saliva samples from students, teachers and school personnel, which are pooled and analyzed together by PCR, making this screening approach quick and cheap. In case of a positive result, samples are re-collected and re-analyzed individually.

In the frame of this project, a pilot study has been already conducted, demonstrating the feasibility and reliability of the method, and in September 2022 the whole study will be launched, which will involve schools in Portugal, Germany, Mexico and Italy, for a total of 440 kindergarten, elementary, intermediate and high school classes.

In this context, we have recently published the results of a meta-analysis [10] showing that students play a significantly minor role in viral spread as compared to teachers. Our analysis is based on SARS-CoV2 infections data from 22 studies, involving over 120.000 subjects including students, teachers and other personnel, from Europe, United States and Israel, and considered results of screenings, contact tracing, and antibody presence. We showed that schools did not increase transmission of COVID-19 but mirrored the infection spread within the community. Moreover, students resulted to be less susceptible to infection and less infective, as infections most frequently occurred between teacher and student.

By the end of this study, we will have identified and characterised the applicability of the LM for SARS-CoV-2 surveillance, as well as the impact of pandemic preventive measures on children.

## Supplementary Information

**Additional file 1.** Specific indications on the materials and procedures are reported.

## Data Availability

Data sharing is not applicable to this article as no datasets were generated or analysed during the current study.

## Abbreviations

AI: Artificail intelligence
LM: Lolli-Methode
NPI: Non-pharmacological intervention
SoC: Standard of care
VoC: Variant of concern
