# Supplementary material for: European Cohorts of patients and schools to Advance Response to Epidemics (EuCARE): a cluster randomised interventional and observational study protocol to investigate the relationship between schools and SARS-CoV-2 infection
Source: BMC Infect Dis. 2023 Jan 3;23:1. doi: 10.1186/s12879-022-07947-6 (PMC9808677; doi:10.1186/s12879-022-07947-6)
Supplement: Supplementary file 1 — Additional file 1. Specific indications on the materials and procedures are reported. [file 12879_2022_7947_MOESM1_ESM.docx]

1. **Materials and Methods**
   1. **Investigational Product and Indication**

We recommend the use of a collection swab with the following characteristics ~2.5mm x ~152mm, Nylon flocked head ~20mm, with intended braking point at ~80mm in individual peel pack sterile (nasopharyngeal swab) in the case of the samples collected for pooling, due to two main reasons: i) the number of swabs that can be added to a single 50ml tube, and ii) the fluid volume (PBS or saline solution) needed to hydrate the pools. In the case of the individual sample collection, the use of swabs with their own dry transport tube is highly recommended, for this case the main recommendations are: i) swab in tube, no medium, 108x16mm, stem material PS, stem length 83mm, swab material viscose, sterile (Starsted cat# 80.625) or ii) swab in tube, no medium, 175x12mm, stem material PS, stem length 113mm, swab material viscose, sterile (Starsted cat# 80.1301).

The collection tubes will be used only to pool, collect, and transport the samples. The tubes are standard laboratory 50ml tubes with following characteristics, dimensions 29,1mm x 114,4mm with screw cap.

PBS or 0,9% NaCl solution is added to the collection tubes to hydrate and wash the swabs. This fluid for the PCR and long-term storage, therefore the PBS will be Ca- and Mg-free and, in case of the use of saline solution, it will be at 0,9%NaCl.

## List of data to be collected for primary outcome

- Number of teachers and workers in the school
- Number of students in each class
- Number of positive cases in each class randomised to Lolli-Method
- Number of positive cases in each class randomised to SoC
- Percentage of classes participating in the interventional study, by time
- Percentage of students participating in the Lolli-Methode, by time

Variables will be collected by questionnaires and CRFs (see [Annex](#bookmark=id.pgvaqkyvwq3j) 1) administered to: school principals, teachers, laboratories.

## List of data to be collected for secondary outcomes

Data will be collected through questionnaires and CRFs and will be administered to school principals, teachers, students, families, and laboratories. All the data collected through the questionnaires will be used for the secondary outcomes.

**Psychological variables**

Questionnaires will be delivered at the beginning and at the end of each study period.

Psychological consequences in terms of emotions and beliefs concerning preventive measures will be evaluated according to age ranges and by adapting items and response options from questionnaires used for previous studies [e.g., 1]. Questions regarding attitude toward vaccinated and non-vaccinated students will be created *ad hoc* for this study and will be targeted at students from middle and high school and teachers from all school orders except for kindergartens. Psychological status will be assessed depending on participants’ age, as follows:

*Children (kindergarten and primary school) and adolescents (middle and high school)*. Emotional and behavioural difficulties will be assessed using the Strengths and Difficulties Questionnaire (SDQ) [2, 3]. Depression and anxiety will be assessed through the 25-item Revised Children’s Anxiety and Depression Scale (RCADS) [4]. Versions for children will be filled in by caregivers, whereas adolescents will complete the questionnaires by themselves.

*Teachers and school staff*. Psychological general well-being will be investigated using the short version of the Psychological General Well-Being Index (PGWBI-S) [5]. Depression and anxiety will be assessed through the corresponding two subscales of the Depression Anxiety Stress Scales (DASS-21) [6].

## Definition and Assessment of safety related events

• Health hazards that require measures: the Lolli procedure is safe, no health hazards are predictable and no health hazards have been ever signalled in the pilot and deployment phases of the Lolli-Methode in Germany in over 3000 schools for 7 months. However, it may in principle happen that a person uses the Lolli incorrectly, inside his/her own mouth or outside, and gets a little hurt. The event will be recorded in the study eCRFs by the teachers and managed following the routine procedures at school for minor accidents.

- Findings in the trial that may affect the safety of study participants and which require preventive or corrective measures intended to protect the health and safety of study participants: none
- Findings which require preventive or corrective measures: none
- Any discomfort following the use of Lolli-Methode will be collected in the eCRFs and regularly reported to the Investigators.

## Reporting of Safety related events

Reporting to the Investigator:

• Health hazards that require measures are reported to the Investigator within 24 hours upon becoming aware of the event.

Reporting to the Authorities:

• In Category A studies it is the Investigator’s responsibility to report to the local Ethics Committee.

• Health hazards that require measures within 2 days.

# Quality assurance and control

## Data handling and record keeping / archiving

### Case Report Forms

Case report forms (CRFs) and electronic CRFs (eCRFs) will be elaborated by EuResist in accordance with the Questionnaires reported in and under the guidance of the involved PIs. eCRFs will be available via computer and mobile telephone interface.

### Specification of source documents

Source documents for the study are:

- Registries of school presence
- Informed consents of the participants
- Contact tracing reports by the healthcare authorities

### Record keeping / archiving

Registries of school presence are kept at the schools following the usual procedures.

Informed consent sheets will be kept at the schools by the school personnel.

Contact tracing reports are kept at the involved healthcare authorities following the usual procedures.

## Data management

### Data Management System

EuResist will develop the eCRFs for data input following the CRFs of the study (realised from the questionnaires and CRFs ) under the supervision of the study PIs. The eCRFs will be accessible online both via PC and via mobile phones.

The data will be inputted into the system by the School Principals (or delegated person), teachers, students, families and by the laboratories for the PCR analysis of the samples.

Data collected via the eCRFs as well as retrospective data integrated by EuResist data managers will be stored in the EuCARE centralised relational database at EuResist. MariaDB relational database management system (RDBMS) will be used both for the collection of data via the eCRF and for the storage of the cleansed data.

The system will also have a results reporting page for the school authorised personnel to see the laboratory results.

Once the database is freezed at the end of the study period and the data has been validated, the pseudonymised data will be provided to the study PIs for analysis. Anonymised data will be provided to IBM Israel for AI based analysis.

### Data security, access and back-up

The database will be hosted on a secure Cloud server, located in the European Union, compliant to the GDPR. Back-up copies of the database and the management procedures will be generated with daily frequency on a different storage location. Access will be restricted to authorised EuResist personnel accounts, authenticated by password, geolocation and access device checks. Track of change logs will be stored.

### Electronic and central data validation

Quality assurance routines will be performed to check consistency, completeness and integrity of the data. Upon alert from the system the data management staff will transmit to the monitor staff of the involved PI (the PI that enrolled the school in question) the issues to be fixed and will follow-up until the complete resolution of the issues.

## Monitoring

The monitoring activity will regard the adherence to the correct procedures at the schools and at the laboratories, including the proper and timely filling of the study eCRFs, as well as the proper administration and collection of the informed consent forms.

Monitoring at each school will be performed by the PI who enrolls the school.

## Confidentiality, Data Protection

Names of the participants and class identifiers are collected and stored in encrypted fashion in the database. Only school’s authorised personnel and the certified involved laboratories have the key to decrypt this information.

For all other users of the system the data are managed in a pseudonymised fashion: each class and each individual is identified by a randomly assigned ID code.

Data will be securely transferred and stored. Every reasonable step will be taken to protect the privacy of participants' health information and to prevent misuse of this information. The participants' record (paper/digital) may be seen by institutional Review Boards (IRBs) or Ethic Committees (ECs) who review the study to make sure it is ethically acceptable and by research staff and study monitors, and their designees.

Data (information) will not be identified by name, or in any other way, in any publication about this study. The participants will be identified only by a code and personal information from study records will not be released without the participant’s written permission.

Participants will be informed about the above conditions in the Informed Consent forms.

## Storage of biological material and related health data

### Sample taken > test

The saliva samples can be transported and stored at room temperature. They will be collected in the classrooms into the falcon tubes and stored in the transport bags at the designated place (school secretary). The designated place may temporarily store the samples until the collection by the courier keeping samples far from sunlight and heat areas. The relevant information will be written on the tube label and copied into the eCRFs.

The results will be sent per email to the health authorities and the responsible of the school to assure the individual sample collection after a positive pool and the correct report to the authorities after a positive individual detection.

### After test

It is not necessary to store the pooled saliva samples and the negative individual samples after the analysis at the labs. However, these samples could be stored for future research purposes.

The individual positive samples will be stored under long storage conditions, namely in 1.5ml tubes at -70° temperature for variant analysis and for possible further studies (whole genome sequencing).

The samples stored for variant analysis will be either analysed onsite by the same laboratory that did the PCR analysis, or shipped to a designated laboratory for variant analysis.

# Publication and dissemination policy

The findings from this study, positive, negative or inconclusive, are intended to be published in peer-reviewed journals and/or presented at national and international conferences. Preference will be given to open access journals.

Authorship on publications will be based on academic standards and custom. In accordance with normal academic practice, all investigators and contributors to a publication will be acknowledged, always in compliance with recognized standards concerning publication and authorship, including the most recent “Recommendations for the Conduct, Reporting, Editing and Publications of Scholarly Work in Medical Journals” developed by the International Committee of Medical Journal Editors (ICMJE).

All publications and presentations will be listed on the EuCARE webpage.

# References

1. Lanciano, Tiziana, et al. "Risk perceptions and psychological effects during the Italian COVID-19 emergency." *Frontiers in psychology* 11 (2020): 580053.
2. Goodman, Robert. "The Strengths and Difficulties Questionnaire: a research note." *Journal of child psychology and psychiatry* 38.5 (1997): 581-586.
3. Goodman, Robert, Howard Meltzer, and Veira Bailey. "The Strengths and Difficulties Questionnaire: A pilot study on the validity of the self-report version." *European child & adolescent psychiatry* 7.3 (1998): 125-130.
4. Chorpita, B. F., Yim, L. M., Moffitt, C. E., Umemoto L. A., & Francis, S. E. Assessment of symptoms of DSM-IV anxiety and depression in children: A Revised Child Anxiety and Depression Scale. Behaviour Research and Therapy 38 (2000): 835-855.
5. Grossi, Enzo, et al. "Development and validation of the short version of the Psychological General Well-Being Index (PGWB-S)." *Health and quality of life outcomes* 4.1 (2006): 1-8.
6. Lovibond, S. H., & Lovibond, P. F. Manual for the Depression Anxiety Stress (2nd ed.). Sydney, New South Wales, Australia: Psychology Foundation (1995).
